# Broadly neutralizing human antibodies against Omicron subvariants of SARS-CoV-2

**DOI:** 10.1186/s12929-023-00955-x

**Published:** 2023-07-31

**Authors:** Hsiao-Ling Chiang, Kang-Hao Liang, Ruei-Min Lu, Ting-Wen Kuo, Yi‑Ling Lin, Han-Chung Wu

**Affiliations:** 1 Biomedical Translation Research Center (BioTReC), Academia Sinica, Taipei, Taiwan; 2 Institute of Cellular and Organismic Biology (ICOB), Academia Sinica, No. 128, Academia Road, Section 2, Nankang, Taipei 11529 Taiwan; 3 Institute of Biomedical Sciences (IBMS), Academia Sinica, Taipei, Taiwan

**Keywords:** Severe acute respiratory syndrome coronavirus 2 (SARS-CoV-2), Omicron, XBB.1.5, BQ.1.1, Single B cell cloning, Neutralizing human antibody

## Abstract

**Background:**

The COVID-19 pandemic continues to pose a significant worldwide threat to human health, as emerging SARS-CoV-2 Omicron variants exhibit resistance to therapeutic antibodies and the ability to evade vaccination-induced antibodies. Here, we aimed to identify human antibodies (hAbs) from convalescent patients that are potent and broadly neutralizing toward Omicron sublineages.

**Methods:**

Using a single B-cell cloning approach, we isolated BA.5 specific human antibodies. We further examined the neutralizing activities of the most promising neutralizing hAbs toward different variants of concern (VOCs) with pseudotyped virus.

**Results:**

Sixteen hAbs showed strong neutralizing activities against Omicron BA.5 with low IC_50_ values (IC_50_ < 20 ng/mL). Among four of the most promising neutralizing hAbs (RBD-hAb-B22, -B23, -B25 and -B34), RBD-hAb-B22 exhibited the most potent and broad neutralization profiles across Omicron subvariant pseudoviruses, with low IC_50_ values (7.7–41.6 ng/mL) and a low PRNT_50_ value (3.8 ng/mL) in plaque assays with authentic BA.5. It also showed potent therapeutic effects in BA.5-infected K18-hACE2 mice.

**Conclusions:**

Thus, our efficient screening of BA.5-specific neutralizing hAbs from breakthrough infectious convalescent donors successfully yielded hAbs with potent therapeutic potential against multiple SARS-CoV-2 variants.

**Supplementary Information:**

The online version contains supplementary material available at 10.1186/s12929-023-00955-x.

## Background

Since the emergence of COVID-19, humanity has faced continual threats from different variants of SARS-CoV-2, including Alpha (B.1.1.7), Beta (B.1.351), Gamma (P.1) and Delta (B.1.617.2). Toward the end of 2021, the Omicron (B.1.1.529) variant was first reported in South Africa, and it was immediately classified as the fifth variant of concern (VOC) by the World Health Organization (WHO) due to its high transmissibility and potential for immune evasion [8]. Within about one year, Omicron became the overwhelmingly dominant variant worldwide, and it has since diversified into several different sublineages [11, 23, 24, 34]. While the BA.5 subvariant of Omicron became the major pandemic variant in the second half of 2022, other Omicron subvariants have raised concern as well. These include two BA.5 sublineages (BF.7 and BQ.1.1), a BA.2 sublineage (BA.2.75.2), and the BA.2 lineage-recombinant XBB.1 [9].

The Omicron variant encodes a spike protein that is significantly different from previous VOCs, with 15 amino acid changes present in the receptor binding domain (RBD). Since the RBD of SARS-CoV-2 plays a crucial role in binding of the virus to the human angiotensin-converting enzyme 2 (ACE2) receptor, these mutations cause Omicron to exhibit a 2.4-fold increase in binding affinity to ACE2. Thus, Omicron is even more infectious than the Delta variant, which was the fourth VOC [4]. Moreover, newly emerging subvariants have continually replaced older Omicron subvariants and raised global concerns about the potential for new outbreaks. Currently, the most commonly circulating Omicron subvariants are BQ.1.1 and XBB.1.5. The BQ.1.1 subvariant is derived from BA.5 and contains additional R346T/K444T/N460K mutations, which confer a 2.6-fold increase in neutralization resistance compared to BA.5 [34]. The other common subvariant, XBB.1, contains nine additional mutations in its RBD compared to BA.2. Furthermore, the XBB.1.5 subvariant of XBB.1 carries an S486P mutation, which increases its usage of ACE2 and leads to more effective immune evasion [16, 51]. Thus, both BQ.1.1 and XBB.1.5 subvariants have increased transmissibility and immune evasion compared to BA.5, and these two viral strains are currently gradually replacing BA.5 as the dominant variants [10, 16, 27, 42]. On April 17, 2023, the WHO classified XBB.1.16 as a variant of interest [45]. This emerging substrain exhibits two additional mutations (E180V and K478R) in its spike protein, as compared to XBB.1.5. Despite these mutations, the cell entry and neutralization sensitivity profiles of XBB.1.16 are similar to those of XBB.1.5 [29]. Therefore, the increased prevalence of XBB.1.16 around the world might be due to mutations outside of the spike protein coding sequence [47].

Monoclonal antibodies (mAbs) are highly specific and versatile tools for basic research and clinical applications [26]. These properties have also allowed mAbs to become essential drugs for combatting COVID-19 [18]. Over the past three years of the SARS-CoV-2 pandemic, single B cell sorting and cloning of human antibodies (hAbs) has been a reliable approach for antibody drug development [14, 15, 20, 32]. Using this approach, it only takes three to four weeks to gather samples and identify lead sequences for further development. In fact, the neutralizing antibody Bamlanivimab (LY-CoV555) was selected by this strategy and produced by GMP manufacturing within only 2 months [20]. The speed of this process allowed Bamlanivimab to become the first SARS-CoV-2 antibody drug to be granted an emergency use authorization (EUA). Other therapeutic antibodies with EUAs were also derived from human single B cells, including Etesevimab, Bebtelovimab, Sotrovimab, Cilgavimab and Tixagevimab. Unfortunately, with the emergence of different Omicron subvariants, most of the therapeutic antibodies that previously received EUAs from the U.S. FDA do not exhibit neutralizing activities toward currently circulating variants [1, 10, 16, 33, 42]. For instance, Bebtelovimab demonstrates strong neutralizing ability against the BA.5 variant of the SARS-CoV-2 virus, but it is ineffective against the BQ.1.1 and XBB.1.5 variants [1, 51]. Among the hAbs with current EUAs, only Sotrovimab has been found to have any neutralizing ability against the XBB.1.5 variant, but its activity is weak (IC_50_ = 915 ng/mL) [51]. Thus, the development of new potent broadly neutralizing antibodies has become an urgent need for combatting SARS-CoV-2.

In our previous work, we identified neutralizing mAbs by utilizing hybridoma technology in combination with lipid nanoparticle (LNP)-encapsulated mRNA immunization of mice, mimicking the recently popularized vaccine strategy [17]. In doing so, we successfully identified mAbs with neutralizing activities against Omicron BA.1 and BA.2 [25]. Although these mAbs can broadly neutralize previous VOCs, we found that they are ineffective at neutralizing the BA.5 variant, an emergent variant at the time. In this study, we sought to quickly identify potent neutralizing antibodies for current Omicron variants using a single B cell cloning method. We isolated Omicron-specific memory B cells from individuals who had experienced breakthrough infections, and then we analyzed the neutralizing potencies of these hAbs toward Alpha, Beta, Gamma, Delta and Omicron variants, including BF.7, BA.2.75.2, BQ.1.1, XBB.1.5, and CH.1.1 sublineages. Several of the hAbs were found to neutralize not only past VOCs but also current variants, which suggests a high potential for clinical utility.

## Materials and methods

### Patients and sample collection

Plasma and peripheral blood mononuclear cells (PBMCs) were isolated from three convalescent patients with COVID-19 symptoms of fever and cough. Blood was collected from the patients in EDTA-containing tubes and separated by centrifugation. The plasma was used for examination of antibody binding and neutralizing abilities, and the PBMCs were isolated for further antigen-specific memory B sorting. All collections were conducted following a protocol that was reviewed and approved by the Institutional Review Board of Biomedical Science Research (IRB-BM) at Academia Sinica (AS-IRB-BM-20006).

### Isolation of PBMCs

The blood in EDTA tubes was diluted with an equal volume of PBS containing 2% FBS. The diluted sample was added to a SepMate™ tube (Stemcell™ technologies), which contained Ficoll-Paque™ Plus (Cytiva). The tube was centrifugated at 800 × *g* for 20 min at room temperature. The plasma was kept for further ELISA or pseudovirus neutralization assays, and the PBMC cells were transferred to a new tube and washed with PBS containing 2% FBS. After counting, the 1–2 × 10^7^ PBMCs were frozen per vial and stored at − 80 °C for 48 h. The cells were transferred to liquid nitrogen for storage.

### RBD-specific B cell sorting

The frozen PBMC cells were thawed at 37 °C and then counted. The cells were stained for 30 min on ice with APC-eFluor 780 (to identify live cells), PE-Cy7 mouse anti-human CD27 (BD Biosciences), BV510 mouse anti-human CD19 (BD Biosciences), and RBD-BV421 (BD Biosciences) or RBD-PE (Abcam) in PBS containing 2% FBS. After washing, the cells were resuspended in PBS with 2% FBS, and RBD-specific memory B cells (CD19 + CD27 + RBD +) were sorted into 96-well PCR plates containing 10 µl capture buffer (10 µl of 1 M Tris–HCl, pH 8.0, and 25 µl of RNasin in 1 mL RNase-free water) via BD FACSAria III (BD Biosciences). The plates were stored at − 80 °C for further experiments.

### Cloning and expression of antibodies

The 96-well plates containing sorted B cells were thawed on the ice and used for RT-PCR (Qiagen OneStep RT-PCR Kit) followed by nested PCR with primers described in a previous study [38]. After analysis of the VDJ sequence by IMGT, the V_H_ and V_K_ fragments were amplified by PCR and subjected to appropriate restriction enzyme digestion. The V_H_ genes were cloned into a modified expression vector with signal peptide and the human constant region of IgG1. The V_K_ genes were cloned into a modified expression vector with a signal peptide and the human kappa chain constant region. The V_H_ and V_K_ plasmids were co-transfected into Expi293F cells (Thermo Scientific) for antibody production. After 5 days of culture, the individual antibodies in the culture supernatant were purified using protein G resin (GE healthcare). The antibodies were replaced into PBS and analyzed by SDS-PAGE.

### ELISA

ELISA plates were coated with 1 µg/mL RBD-His of SARS-CoV-2 WT or different variants in coating buffer (0.1 M NaHCO_3_, pH 8.6) at 4 °C overnight, followed by washing with PBS and blocking with 1% BSA in PBS at room temperature for 2 h. After blocking, different concentrations of antibodies or expression medium were added to the wells for 1 h at room temperature; human serum was diluted in 3% skim milk in PBS. The plates were washed with PBST_0.1_ (PBS containing 0.1% Tween-20) three times, and then horseradish peroxidase-conjugated anti-human IgG (Jackson ImmunoResearch) (1:5,000) was added and incubated for 1 h at room temperature. After three washes with PBST_0.1_, the signal was developed using TMB solution (TM1999, Scytek Laboratories). The reaction was stopped with TMB Stop Buffer (TSB999, Scytek Laboratories), and absorbance was measured at 450 nm by an ELISA reader (Versa Max Tunable Microplate Reader; Molecular Devices).

### Pseudovirus neutralization assay

Pseudovirus neutralization assays were performed using HEK293T cells with stable expression of human ACE2 (HEK293T/hACE2). The different variants of SARS-CoV-2 full-length spike protein were expressed from pseudotyped lentiviruses provided by the National RNAi Core Facility (Academia Sinica, Taiwan). The HEK293T/hACE2 cells were seeded in 96-well white plates (Corning Costar) at a density of 1 × 10^4^ cells per well, and cultured at 37 °C for 24 h. Various concentrations of antibodies were pre-incubated with different SARS-CoV-2 variant pseudoviruses at 1000 TU/well in the 96-well plates at 37 °C for 1 h. Then, the mixtures were added to pre-seeded HEK293T/hACE2 cells. After 24 h incubation at 37 °C, the supernatants were replaced with DMEM + 10% FBS for an additional 48 h. Next, ONE-Glo™ luciferase reagent (Promega) was added to each well for 3 min. Luminescence was measured with a microplate spectrophotometer (Molecular Devices). The inhibition rates of 0% or 100% were respectively defined by wells with only pseudovirus or cells. The half-maximal inhibitory concentration (IC_50_) was calculated by nonlinear regression using Prism software version 9 (GraphPad Software Inc.). The average IC_50_ value for each antibody was determined from at least two independent experiments.

### Cellular ELISA

HEK293T cells were transiently transfected with wild-type or mutant RBD plasmids in 6-well plates. The next day, the cells were seeded in 96 well-plates. The cells were fixed in 4% paraformaldehyde in PBS for 15 min at room temperature at 48 h after transfection. Fixed cells were incubated in 0.1% Triton X-100 at room temperature for 10 min. After blocking with 5% milk, antibodies against RBD were added to the wells (1 µg/mL) for 1 h at room temperature. After washing, HRP-conjugated anti-human antibody (Jackson ImmunoResearch) (1:2000) was added for 1 h at room temperature. After washing off the excess secondary antibody, the signal was produced using TMB solution (TM1999). The reaction was stopped with TMB Stop Buffer (TSB999), and absorbance was measured at 450 nm by an ELISA reader (VersaMax Tunable Microplate Reader).

### Western blotting analysis

A total of 40 ng recombinant BA.5-RBD or -spike protein with a polyhistidine tag was analyzed in either reducing or non-reducing conditions. Each sample was separated by SDS-PAGE and transferred to a PVDF membrane, followed by hybridization with RBD-hAbs (1 µg/mL), normal human IgG or anti-His antibody. The blot was then hybridized with HRP-conjugated anti-human immunoglobulin and developed with enhanced chemiluminescence reagents.

### Authentic virus neutralization assay

Omicron BA.5 (hCoV-19/Taiwan/689423/2022) was used for a live virus plaque reduction neutralization test (PRNT). The experiments were performed at the BSL-3 facility in the Institute of Biomedical Sciences, Academia Sinica. RBD-hAbs were serially diluted in PBS and pre-incubated with 100 plaque-forming units (PFU) SARS-CoV-2 BA.5 for 1 h at 37 °C. The mixtures were then added to pre-seeded Vero-E6 cells for 1 h at 37 °C. The virus-containing culture medium was removed and replaced with DMEM containing 2% FBS and 1% methyl-cellulose for an additional 4-day incubation. Cells were then fixed with 10% formaldehyde and stained with 0.5% crystal violet for 20 min. The plates were washed with distilled water and plaque number formed at each dilution were counted. The 50% plaque reduction (PRNT_50_) values were calculated with Prism software.

### In vivo therapeutic assay in SARS‑CoV‑2 BA.5-infected mice

The K18-hACE2 transgenic mouse model of SARS-CoV-2 BA.5 infection was used to evaluate the therapeutic potency of RBD-hAb-B22. All animal studies were approved by and performed according to the guidelines of the Institutional Animal Care and Use Committee (IACUC) of Academia Sinica, Taiwan (protocol 22-11-1921). Each mouse was intranasally inoculated with 10^4^ PFU SARS-CoV-2 BA.5 (hCoV-19/Taiwan/TSGH-8189/2022), and RBD-hAb-B22 was intraperitoneally injected into mice at day 2 after virus inoculation. The mice were sacrificed for collection of lung tissue at day 3 post-inoculation. The tissue was homogenized and the supernatant was used for the TCID_50_ assay (50% tissue culture infectious dose). Homogenates were serially diluted and applied to a Vero-E6 cell monolayer in DMEM with 1% FBS for 4 days. The cytopathic effect (CPE) was detected and used to calculate the TCID_50_, i.e., the amount of a virus required to infect 50% of inoculated cells.

## Results

### Derivation of anti-SARS-CoV-2-BA.5 neutralizing human antibodies (hAbs) from single B cells of convalescent donors

**Fig. 1:**
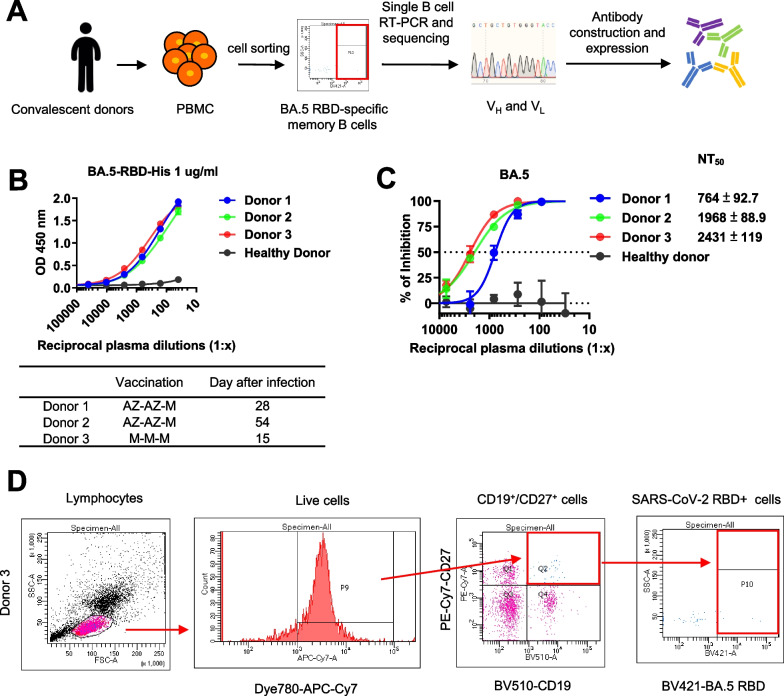
Isolation of memory B cells targeting SARS-CoV-2 BA.5-RBD from convalescent donors. **A** Overview of experimental design. CD19^+^CD27^+^ BA.5-RBD^+^ B cells from convalescent donors were sorted by flow cytometry. The V_H_ and V_L_ fragments in single B cells were amplified and sequenced, and the resulting constructs were transfected to Expi293F cells for antibody production. **B** Determination of the binding activity of donor plasma by ELISA. The plasma was incubated in ELISA plates coated with 1 µg/mL recombinant BA.5-RBD protein, followed by detection with HRP-conjugated anti-human antibody. The table below shows the types and orders of vaccinations received by the convalescent donors and the day of blood sample collection after breakthrough infection. AZ: AstraZeneca COVID-19 vaccine. M: Moderna COVID-19 (mRNA-1273) vaccine. **C** Neutralization assay of SARS-CoV-2 BA.5 pseudovirus with the three convalescent donors. The neutralizing titer 50 (NT_50_), indicating the plasma dilution resulting in half-maximal inhibition was calculated. **D** The gating strategy for BA.5-RBD-specific memory B cell from donor 3. From left to right, the panels show gatings used to isolate lymphocytes, live cells, BV-510-CD19^+^PE-Cy7-CD27^+^ cells (Q2 population), and CD19^+^CD27^+^ cells, which were further gated for BV421-BA.5-RBD

**Fig. 2:**
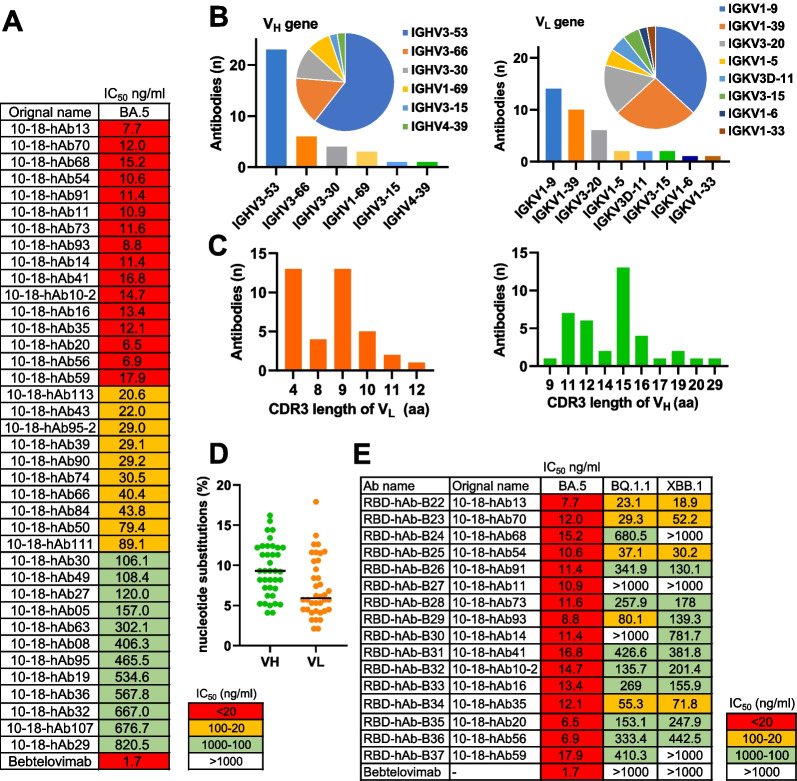
Characteristics of 38 SARS-CoV-2 Omicron BA.5-neutralizing antibodies derived from memory B cells of convalescent donors. **A** Heatmap shows the pseudovirus neutralization activities for the subset of antibodies (n = 38) with observable neutralizing activities (IC_50_ < 1000 ng/mL) against Omicron BA.5. The colors indicate the range of IC_50_: red, < 20 ng/mL; yellow, 20–100 ng/mL; green 100–1000 ng/mL. **B** The gene family usages of V_H_ and V_L_ for the 38 identified neutralizing antibodies against BA.5-RBD protein. **C** The amino acid lengths of the CDR3 loop of V_H_ and V_L_ for the 38 antibodies. **D** Rates of nucleotide substitutions in V_H_ and V_L_ for the 38 antibodies. **E** Pseudovirus neutralization activities of the 16 most potent antibodies (IC_50_ < 20 ng/mL) against Omicron BA.5, BQ.1.1 and XBB.1 subvariants. The 16 antibodies were renamed sequentially from RBD-hAb-B22 to RBD-hAb-B37

To rapidly generate hAbs against RBD SARS-CoV-2-BA.5, we performed RT-PCR on single B cells from convalescent patients [38], as outlined in Fig. 1A. Plasma and PBMCs were collected from three COVID-19 convalescent donors, who had each received three vaccinations but still experienced breakthrough infections with SARS-CoV-2 Omicron. The donors showed symptoms of fever and cough at some time between 10 June 2022 and 7 October 2022. Using blood samples from the three donors, we analyzed plasma binding by ELISA (Fig. 1B). The plasma from all three donors showed binding to SARS-CoV-2 BA.5 RBD. The plasma neutralizing abilities for SARS-CoV-2 BA.5 were then analyzed by pseudovirus assays (Fig. 1C). The plasma sample of donor 3 exhibited more potent neutralizing activity (NT_50_ = 1/2431) than the samples from donor 1 (NT_50_ = 1/764) and donor 2 (NT_50_ = 1/1968). Therefore, we chose to use the blood sample of donor 3 for isolation of neutralizing antibodies. Using flow cytometry-based cell sorting, CD19 and CD27 double-positive and SARS-CoV-2 BA.5 RBD-bound memory B cells were isolated (Fig. 1D). The genes of heavy chain (V_H_) and light chain (V_L_) variable regions of selected single B cells were amplified with specific primers [38], and the VDJ sequences were analyzed by IMGT. From this analysis, we identified 126 antibody clones and generated each one in Expi293F cells. We then screened the binding and neutralizing abilities of all 126 hAbs by using conditioned media in ELISA and pseudovirus assays. Furthermore, the RBD-hAbs were purified and their abilities to neutralize BA.5 were tested (Additional file 1: Fig. S1A). Among the candidates, 38 hAbs were found to neutralize BA.5 with IC_50_ < 1000 ng/mL, and 16 hAbs showed high neutralizing activities, with IC_50_ < 20 ng/mL (Fig. 2A).

### Characteristics of neutralizing antibodies for SARS-CoV-2 Omicron BA.5 derived from single memory B cells

We conducted a comprehensive analysis of the Ig gene family usage among the 38 neutralizing hAbs and found that six V_H_ gene families were represented, including IGHV3-53 (60.53%), IGHV3-66 (15.79%), IGHV3-30 (10.53%), IGHV1-69 (7.89%), IGHV3-15 (2.63%) and IGHV4-39 (2.63%) (Fig. 2B). These V_H_ genes have also been observed in memory B cells of individuals with breakthrough infections of BA.1 [21]. The three most highly represented V_L_ gene families were IGKV1-9 (36.8%), IGKV1-39 (26.3%) and IGKV3-20 (15.8%). The CDR3 lengths were predominantly 15 amino acids of V_H_ and either 4 or 9 amino acids of V_L_ (Fig. 2C). The somatic hypermutation (SHM) rates of the 38 neutralizing antibodies ranged from 4.1 to 16.2%, with a median of 9.3% (Fig. 2D). Thus, our data indicated that breakthrough infections elicited a higher hypermutation rate of neutralizing hAbs than earlier SARS-CoV-2 variant infections, which induced lower levels of SHM antibodies [30, 53].

We renamed the 16 neutralizing hAbs with IC_50_ < 20 ng/mL as RBD-hAb-B22 to RBD-hAb-B37. Then, we evaluated their neutralizing potencies toward current clinically relevant variants, BQ.1.1 and XBB.1 (Fig. 2E and Additional file 1: Fig. S1B and C). Pseudovirus assays showed that four of the 16 hAbs, i.e., RBD-hAb-B22, -B23, -B25 and -BF34, could broadly and potently neutralize BQ.1.1 and XBB.1 with IC_50_ values ranging from 18.9 to 71.8 ng/mL.

### Binding activities and epitopes of RBD-hAbs across selected SARS-CoV-2 variants

**Fig. 3:**
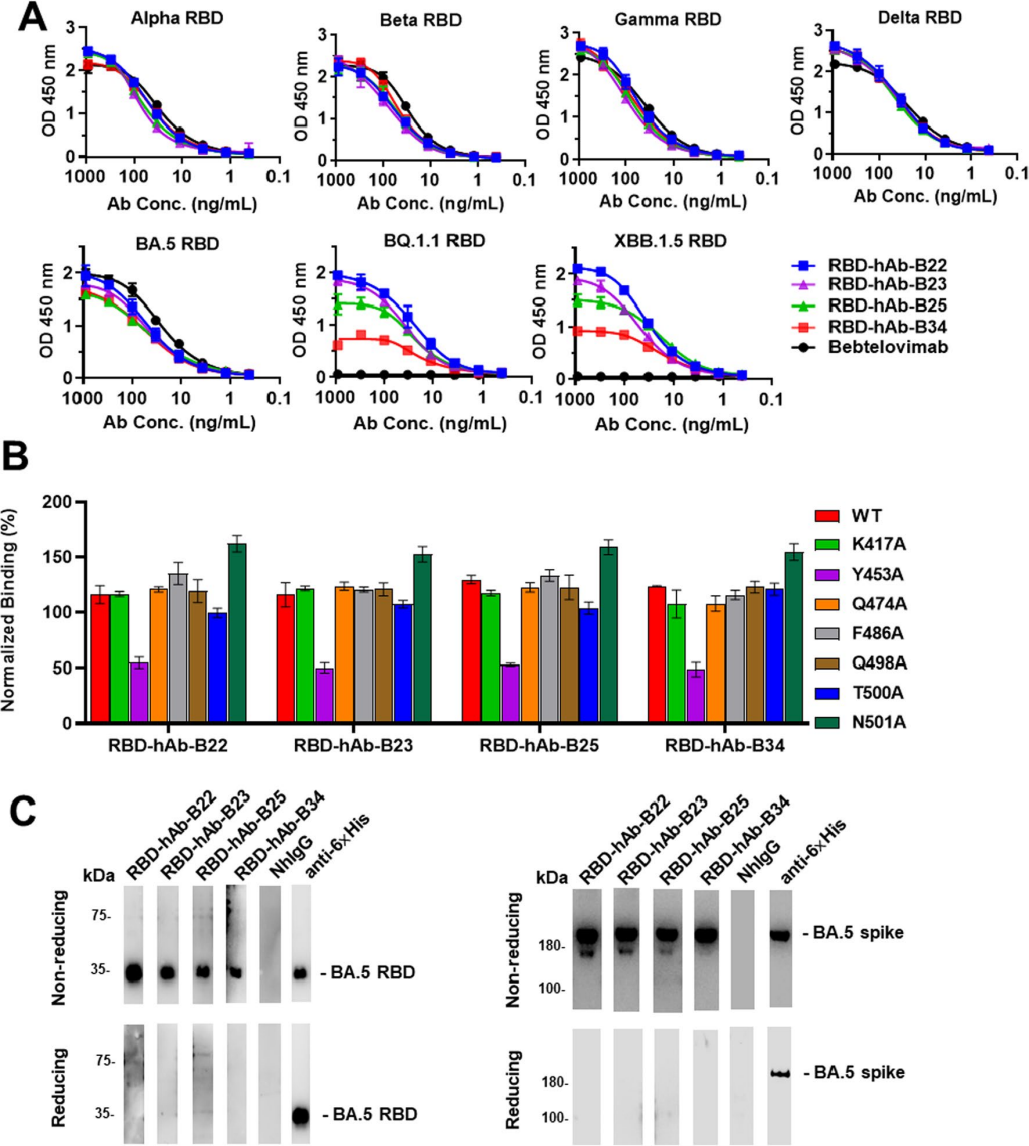
Examination of RBD-binding abilities and epitope mapping of RBD-hAb-B22, -B23, -B25 and -B34 antibodies. **A** Binding activities of four RBD-hAbs were measured against RBD proteins of Alpha, Beta, Gamma, Delta, Omicron BA.5, BQ.1.1 and XBB.1.5 by ELISA. Abs were threefold serially diluted from 900 to 0.4 ng/mL. **B** Epitope mapping by mutagenesis assays. HEK293T cells transiently expressing exogenous wild-type (WT) or mutant RBD proteins with single or combinatorial alanine mutations. Binding of the four RBD-hAbs to RBD mutants was examined by cellular ELISA. The results were normalized and are presented as percentages. **C** The four RBD-hAbs were used as primary antibodies for detection of recombinant BA.5 RBD and spike protein-His in Western blots. Anti-6 × His mAb was used as a positive control. Normal human IgG (NhIgG) was used as a negative control

To investigate whether the four RBD-hAbs share overlapping epitopes, we carried out ELISAs with RBD proteins harboring mutations corresponding to selected variants (Fig. 3A). When tested against Alpha, Beta, Gamma, Delta and BA.5, the binding curves of RBD-hAb-B22, -B23, -B25 and -B34 were similar with Bebtelovimab. However, when tested against BQ.1.1 and XBB.1.5, RBD-hAb-B22 exhibited stronger binding than the other three hAbs, while Bebtelovimab showed no detectable binding. The binding curves of RBD-hAb-B34 exhibited similar trends toward decreased affinity when tested against the RBDs of BQ.1.1 and XBB.1.5.

Because seven key RBD residues (K417, Y453, Q474, F486, Q498, T500 and N501) are known to directly interact with the ACE2 receptor [48], we tested whether these residues are involved in the binding of our four RBD-hAbs. Each residue was individually mutated to alanine, and the mutant RBDs were transiently expressed in 293T cells. Then, we performed a series of cellular ELISAs to determine the impact of each residue on binding of the four RBD-hAbs (Fig. 3B). The results showed that the single Y453A mutation dramatically decreased the binding of all four RBD-hAbs by approximately 50%. Mutations at K417, F486 and Q498, which are associated with Omicron subvariants, did not decrease binding potency for any of the four tested RBD-hAbs. In addition, Western blotting analysis showed that four RBD-hAbs could recognize recombinant Omicron BA.5 RBD protein and spike protein at the expected molecular weights in non-reducing conditions but not in reducing conditions (Fig. 3C). These data suggest that the epitopes recognized by the four RBD-hAbs in RBD are very similar and are likely to be structural rather than linear epitopes.

### Potent and broad neutralizing activities of RBD-hAb-B22, -B23, -B25 and -B34 toward SARS-CoV-2 variants

**Fig. 4:**
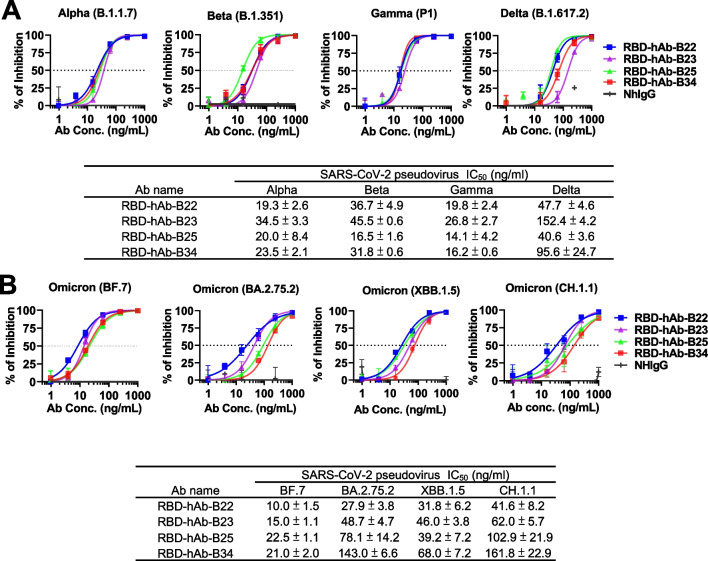
Neutralizing capacities of RBD-hAb-B22, -B23, -B25 and -B34 antibodies toward SARS-CoV-2 variant pseudoviruses. **A** Neutralization activities of RBD-hAb-B22, -B23, -B25 and -B34 against pseudoviruses of previous VOCs, including Alpha, Beta, Gamma and Delta (data are presented as mean ± SE). Representative data from two independent experiments are shown. **B** Neutralization activities of RBD-hAb-B22, -B23, -B25 and -B34 against pseudoviruses of different Omicron subvariants, BA.5, BQ.1.1, XBB.1, XBB.1.5, BF.7, BA.2.75.2, and CH.1.1 (data are presented as mean ± SE). Representative data from two independent experiments are shown

Although the RBD-hAb-B22, -B23, -B25, and -B34 were initially selected according to their abilities to neutralize Omicron BA.5, we were curious whether the RBD-Abs retained potency against other SARS-CoV-2 variants. Therefore, we examined the broad neutralizing abilities of the four RBD-hAbs in pseudovirus assays with four previous VOCs, including Alpha (B.1.1.7), Beta (B.1.351), Gamma (P1) and Delta (B.1.617.2) (Fig. 4A). The results demonstrated that each of the four RBD-hAbs possessed neutralizing ability for all previous VOCs, with IC_50_ values ranging from 14.1 to 152 ng/mL. For Alpha (B.1.1.7), Beta (B.1.351), and Gamma (P1) variants, each of the four RBD-hAbs exhibited potent neutralizing abilities with IC_50_ values under 50 ng/mL. When tested against subvariants of Omicron (BF.7, BA.2.75.2, XBB.1.5 and CH.1.1), the four hAbs also demonstrated broad neutralizing abilities with IC_50_ values ranging from 10 to 161.8 ng/mL (Fig. 4B). Notably RBD-hAb-B22 had the lowest IC_50_ values for Omicron variants, ranging from 10 to 41.6 ng/mL. Based on the results of these ELISA and neutralizing assays, we concluded that RBD-hAb-B22 shows strong and consistent binding ability across all tested variants, and it has potent neutralization ability toward currently circulating Omicron subvariants.

**Fig. 5:**
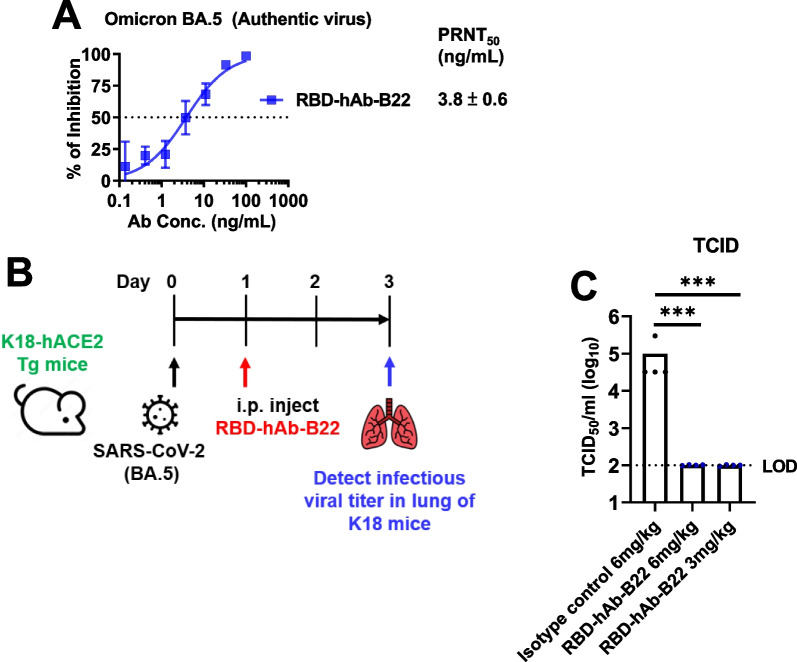
Neutralization ability and in vivo therapeutic assays of SARS-CoV-2 BA.5 of RBD-hAb-B22. **A** Neutralizing RBD-hAb-B22 antibody inhibits SARS-CoV-2 BA.5 variant according to PRNT assay. The PRNT_50_ value was calculated with Prism software (data are presented as mean ± SD). **B** Schematic of the design for neutralizing RBD-hAb-B22 antibody against SARS-CoV-2 BA.5. One day after intranasal (i.n.) infection of SARS-CoV-2 BA.5, the K18-hACE2 mice were given a single intraperitoneal injection of either RBD-hAb-B22 3 mg/kg (n = 4), RBD-hAb-B22 6 mg/kg (n = 4), or normal human IgG control 6 mg/kg (n = 4). On day 3 after virus inoculation, lung samples were collected for analysis. **C** The viral loads in the lungs of treated mice were determined as median tissue culture infectious dose per mL (TCID_50_/mL). *p* values were calculated by two-tailed Student’s *t* test. *p < 0.05 ***p < 0.001

RBD-hAb-B22 generally exhibited lower IC_50_ values and better neutralizing activities than the other three antibodies for all variants. Therefore, we further evaluated the neutralizing potential of RBD-hAb-B22 by conducting an in vitro plaque reduction neutralization test (PRNT) against authentic Omicron BA.5. In this test, RBD-hAb-B22 displayed high potency, with a PRNT_50_ value of 3.8 ng/mL (Fig. 5A). We next examined the therapeutic effect of RBD-hAb-B22 in a SARS-CoV-2 BA.5-infected K18-hACE2 mouse model (Fig. 5B). The K18-hACE2 mice were treated with RBD-hAb-B22 at 1 day post-inoculation with BA.5. The mice were sacrificed at 3 days post-infection, and infectious titers were determined from lung tissue. The infectious BA.5 titers were close to the limit of detection (LOD, 1 × 10^2^ TCID_50_/mL) for 3 mg/kg and 6 mg/kg groups at 3 days post-infection (Fig. 5C). Thus, we concluded that RBD-hAb-B22 exerts potent therapeutic effects in SARS-CoV-2 BA.5-infected K18-hACE2 mice.

## Discussion

Therapeutic mAbs can be generated by four major strategies, including mouse hybridoma, phage-display, hAb transgenic mice, and single B cell cloning [26]. Among these strategies, the single B cell cloning approach has been the most widely used for generating SARS-CoV-2-neutralizing antibodies. In our study, we performed single B cell cloning using blood from a subject who had received three doses of the Moderna mRNA vaccine and subsequently experienced a breakthrough infection. This individual had potent neutralizing plasma, and the cloning yielded 38 neutralizing antibodies. We observed that these antibodies contained higher levels of SHM in the V_H_ gene (Fig. 2D) than antibodies derived in earlier studies. Typically, the RBD-specific neutralizing antibodies isolated during early convalescence exhibit very limited or even no SHM [22, 30, 37, 53]. However, recent reports indicate that the neutralizing antibodies from individuals with breakthrough infections or those that received three doses (but not two doses) of mRNA vaccines tend to show high SHM levels [21, 39, 44], high potency and cross-neutralizing activity [7, 19, 28, 31, 35]. Thus, the PBMCs from individuals who received three-dose mRNA immunizations and breakthrough infections may be a promising source for the discovery of broadly neutralizing antibodies.

Upon analyzing the sequences of our neutralizing antibodies, we observed that more than 85% of the antibodies were derived from the IGHV3-53, IGHV3-66 or IGHV3-30 V_H_ germline genes. Notably, the sequences of all 16 identified potent BA.5-neutralizing hAbs (IC_50_ < 20 ng/mL) contained the IGHV3-53 and IGHV3-66 gene families (Additional file 2: Fig. S2). This result may be expected, as antibodies that target SARS-CoV-2 RBD frequently utilize IGHV3-53 and IGHV3-66 [36, 43, 46], which differ by only one amino acid within framework region 1 (I12 in IGHV3-53 and V12 in IGHV3-66). Moreover, IGHV3-53/66 antibodies generally share similar binding sites within the RBD [3, 13, 50] and are considered class I antibodies, which only recognize the “up” conformation of the RBD [2]. Class I antibodies tend to contain low SHM and short CDR3 regions, which make minor contributions to the antibody interaction profile. Consequently, germline-encoded residues dominate the binding interaction, allowing for potent neutralization to occur with minimal affinity maturation. This characteristic is particularly useful in fighting infectious diseases, as it facilitates a rapid immune response.

Unfortunately, many class I antibodies exhibit poor cross-neutralizing ability toward recent SARS-CoV-2 variants due to the presence of K417N, E484K and N501Y mutations in the RBD [12, 49, 52]. Moreover, almost all previously developed class I antibodies show poor neutralizing ability against Omicron. In contrast, the four antibodies generated in this study display broad cross-neutralizing ability (Fig. 4). Recent studies suggest that certain somatic mutations in IGHV3-55/66 (i.e., F27 to I, L or V, and Y58 to F) may increase binding affinity to the RBD [40, 41]. Correspondingly, approximately half of our 38 antibodies exhibited F27 and Y58 mutations, which may contribute to their high affinities. The most potent and broadly neutralizing antibody we identified, RBD-hAb-B22, exhibited a high somatic mutation rate (11.3%) and contained F27Y/Y58F mutations. Collectively, these data are consistent with previous studies and suggest that B cells in SARS-CoV-2 convalescent patients may undergo affinity maturation, accumulate SHM, and produce antibodies with improved potency and breadth.

According to recent reports, all SARS-CoV-2 mAbs with EUAs exhibit poor neutralizing ability for BQ.1.1 and XBB.1.5 [1]. Only Sotrovimab retains weak neutralizing ability (IC_50_ = 915 ng/mL) against XBB.1.5 [51]. It also appears that a cocktail of SA55 (BD55-5514) plus SA58 (BD55-5840) can broadly and potently neutralize current Omicron variants [6], and SA55 (not SA58) can neutralize BQ.1.1 and XBB.1.5 [5, 51]. However, it remains unclear whether SA55 can also neutralize all four previous VOCs. Antibodies with cross-neutralization ability toward all VOCs and current Omicron subvariants are extremely rare.

## Conclusions

In this study, we identified several potent broadly neutralizing antibodies which can neutralize all five VOCs as well as multiple current Omicron subvariants. Broadly neutralizing antibodies hold great promise as therapeutic tools, and our results provide valuable information regarding the successful creation of broadly neutralizing vaccines and new antibody therapies for COVID-19. Overall, this study contributes to and highlights the importance of ongoing research into the development of broadly neutralizing antibodies for treatment of COVID-19.

## Supplementary Information

**Additional file 1: Figure S1.** Neutralizing capacities of RBD-hAbs toward SARS-CoV-2 variant pseudoviruses. (A) Neutralization curves from one independent experiment with BA.5 in the first screening. (B) Neutralization curves from one independent experiment with RBD-hAb-B22 to RBD-hAb-B37 in the BQ.1.1 cross-reactivity screening. (C) Neutralization curves from one independent experiment with RBD-hAb-B22 to RBD-hAb-B37 in the XBB.1 cross-reactivity screening.

**Additional file 2: Figure S2.** Characteristics of 16 potent neutralizing antibodies for SARS-CoV-2 Omicron BA.5. (A) The gene family usage of V_H_ and V_L_ among 16 potent neutralizing antibodies against BA.5-RBD protein. (B) The amino acid lengths of the CDR3 loops of V_H_ and V_L_ for the 16 antibodies. (C) Rates of nucleotide substitutions in V_H_ and V_L_ for the 16 antibodies.

## Data Availability

All materials and supporting data will be made available upon reasonable request to the corresponding author.

## Abbreviations

COVID-19: Coronavirus disease 2019
SARS-CoV-2: Severe acute respiratory syndrome coronavirus 2
RBD: Receptor-binding domain
ACE2: Angiotensin-converting enzyme 2
VOC: Variant of concern
hAb: Humanized antibody
IC_50_: Half maximal inhibitory concentration
LOD: Limit of detection
EUA: Emergency use authorization
FDA: Food and Drug Administration
SHM: Somatic hypermutation
PRNT: Plaque reduction neutralization test
TCID_50_: Median tissue culture infectious dose
