# Supplementary figures and images for: Broadly neutralizing human antibodies against Omicron subvariants of SARS-CoV-2
Source: J Biomed Sci. 2023 Jul 31;30:59. doi: 10.1186/s12929-023-00955-x (PMC10388472; doi:10.1186/s12929-023-00955-x)

Figure S1

A

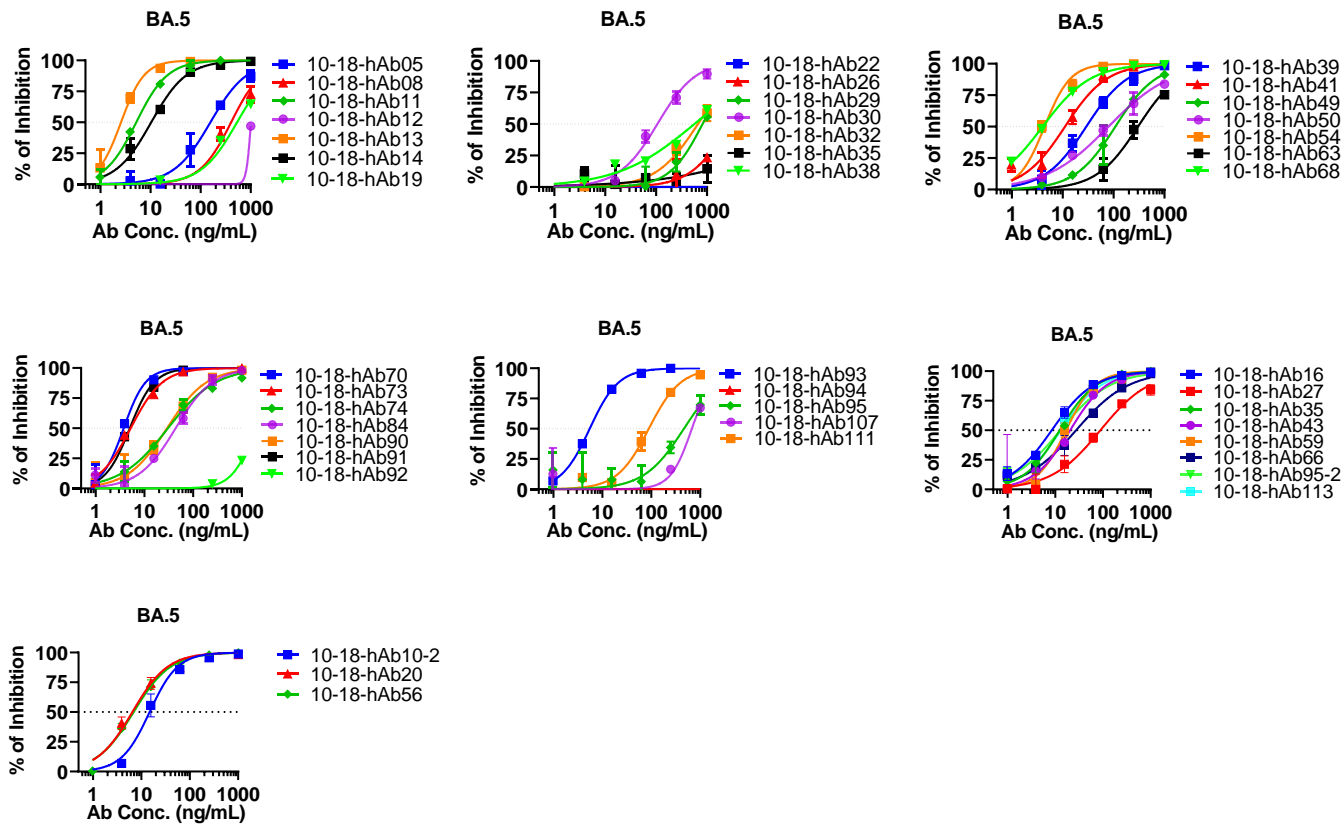

B

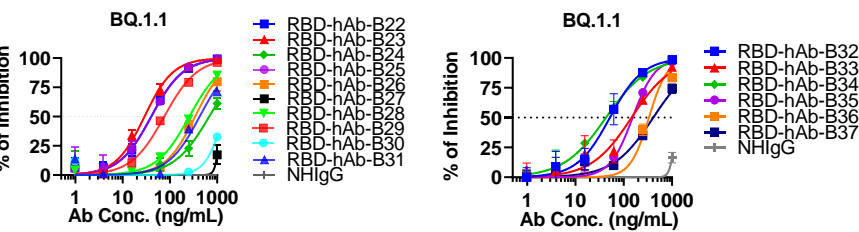

C

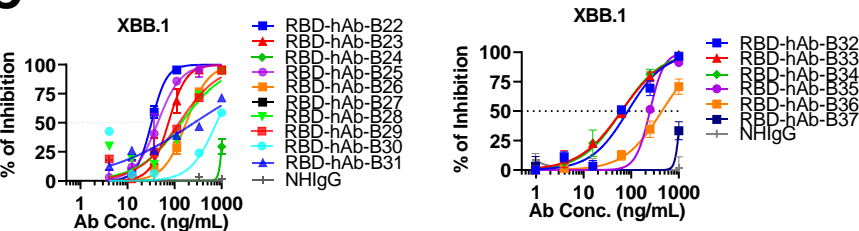

Supplement: Supplementary file 1 — Additional file 1: Figure S1. Neutralizing capacities of RBD-hAbs toward SARS-CoV-2 variant pseudoviruses. (A) Neutralization curves from one independent experiment with BA.5 in the first screening. (B) Neutralization curves from one independent experiment with RBD-hAb-B22 to RBD-hAb-B37 in the BQ.1.1 cross-reactivity screening. (C) Neutralization curves from one independent experiment with RBD-hAb-B22 to RBD-hAb-B37 in the XBB.1 cross-reactivity screening. [file 12929_2023_955_MOESM1_ESM.pdf]

Figure S2

A

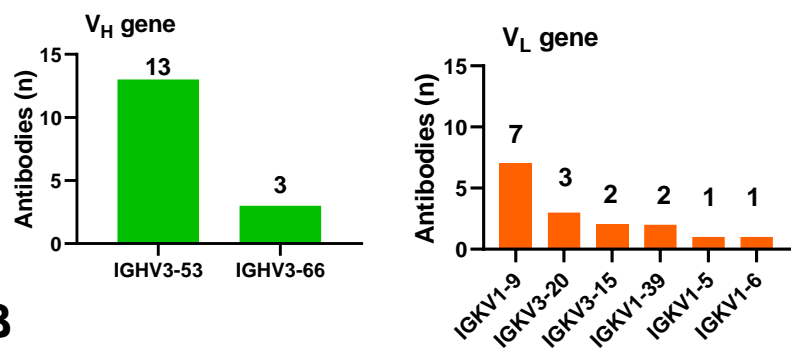

B

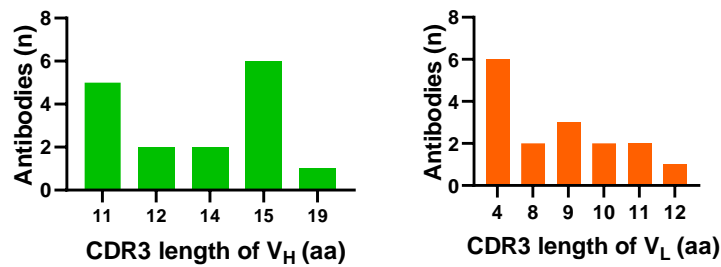

C

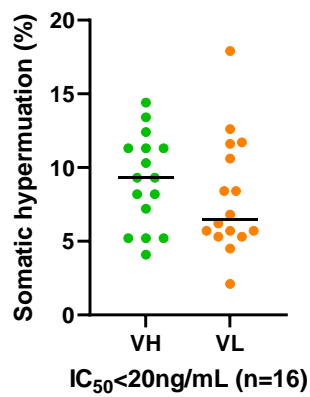

Supplement: Supplementary file 2 — Additional file 2: Figure S2. Characteristics of 16 potent neutralizing antibodies for SARS-CoV-2 Omicron BA.5. (A) The gene family usage of VH and VL among 16 potent neutralizing antibodies against BA.5-RBD protein. (B) The amino acid lengths of the CDR3 loops of VH and VL for the 16 antibodies. (C) Rates of nucleotide substitutions in VH and VL for the 16 antibodies. [file 12929_2023_955_MOESM2_ESM.pdf]
